# The curious case of IDH mutant acute myeloid leukaemia: biochemistry and therapeutic approaches

**DOI:** 10.1042/BST20230017

**Published:** 2023-08-01

**Authors:** Emily Gruber, Lev M. Kats

**Affiliations:** Peter MacCallum Cancer Centre and the Sir Peter MacCallum Department of Oncology, University of Melbourne, Melbourne, VIC 3000, Australia

**Keywords:** 2-HG, acute myeloid leukaemia, IDH mutation, targeted therapies

## Abstract

Of the many genetic alterations that occur in cancer, relatively few have proven to be suitable for the development of targeted therapies. Mutations in isocitrate dehydrogenase (IDH) 1 and -2 increase the capacity of cancer cells to produce a normally scarce metabolite, D-2-hydroxyglutarate (2-HG), by several orders of magnitude. The discovery of the unusual biochemistry of IDH mutations spurred a flurry of activity that revealed 2-HG as an ‘oncometabolite’ with pleiotropic effects in malignant cells and consequences for anti-tumour immunity. Over the next decade, we learned that 2-HG dysregulates a wide array of molecular pathways, among them a large family of dioxygenases that utilise the closely related metabolite α-ketoglutarate (α-KG) as an essential co-substrate. 2-HG not only contributes to malignant transformation, but some cancer cells become addicted to it and sensitive to inhibitors that block its synthesis. Moreover, high 2-HG levels and loss of wild-type IDH1 or IDH2 activity gives rise to synthetic lethal vulnerabilities. Herein, we review the biology of IDH mutations with a particular focus on acute myeloid leukaemia (AML), an aggressive disease where selective targeting of IDH-mutant cells is showing significant promise.

## Introduction

The metabolic enzymes IDH1 and -2 catalyse the interconversion of isocitrate and α-KG via the balancing redox reaction involving NADP^+^ and NADPH. Somatic mutations in *IDH1* and *-2* genes in cancer were first reported in the late 2000's [1] and over the past 15 years enormous strides have been made in our understanding of their contribution to oncogenesis. We now know that these lesions are common in a specific subset of seemingly unrelated malignancies including AML, angioimmunoblastic T cell lymphoma (AITL), glioma, chondrosarcoma and cholangiocarcinoma [2,3]. Hotspot mutations in IDH1 or -2 bestow on the encoded proteins a neomorphic capacity to produce the ‘oncometabolite’ D-2-hydroxyglutarate (2-HG; also known as R-2-hydroxygluterate) [4,5]. 2-HG accumulates to millimolar concentrations in IDH-mutant cells, deregulating a multitude of α-KG-dependent enzymes that include epigenetic, epitranscriptomic and metabolic factors [2,3]. 2-HG, combined with a loss of wild-type IDH function and in collaboration with other genetic events ultimately promotes transformation. Herein we review the genetics, biochemistry, and cell biology of IDH mutations in AML and discuss strategies for targeted therapeutic intervention. We focus specifically on molecular mechanisms rather than the development of specific compounds and observations from clinical studies which have been reviewed in detail elsewhere [3,6,7].

## Biology of IDH mutations in AML

### Enzymatic activity of wild-type and mutant IDH proteins

In mammals, there are three isoforms of IDH encoded by distinct genes with different biochemistry and cellular localisation. IDH1 and -2 are structurally and evolutionary related NADP-dependent homodimers while IDH3 is a divergent NAD-dependent heterotetramer composed of three different subunits. IDH1 is localised predominantly in the cytoplasm and peroxisomes, while IDH2 and -3 are confined within the mitochondrial matrix. The function of IDH3 is thought to be predominantly in mitochondrial respiration, producing α-KG and NADH in one of the irreversible and rate-limiting steps of the TCA cycle. In contrast, the reactions catalysed by IDH1 and 2 are reversible. The ‘forward’ reaction that produces α-KG is also a major source of NADPH, a crucial molecule that provides reducing power for biochemical reactions and protects cells from oxidative stress [8]. When operating in ‘reverse’, IDH1 and -2 contribute to reductive glutamine metabolism, allowing cells to produce citrate and acetyl-CoA to support lipid metabolism and cellular growth in hypoxia [9–11]. The presence of two distinctly localised isoforms allows for isocitrate generated by IDH1 in the cytoplasm to be utilised by IDH2 to generate NADPH in the mitochondria [12].

**Figure 1.**
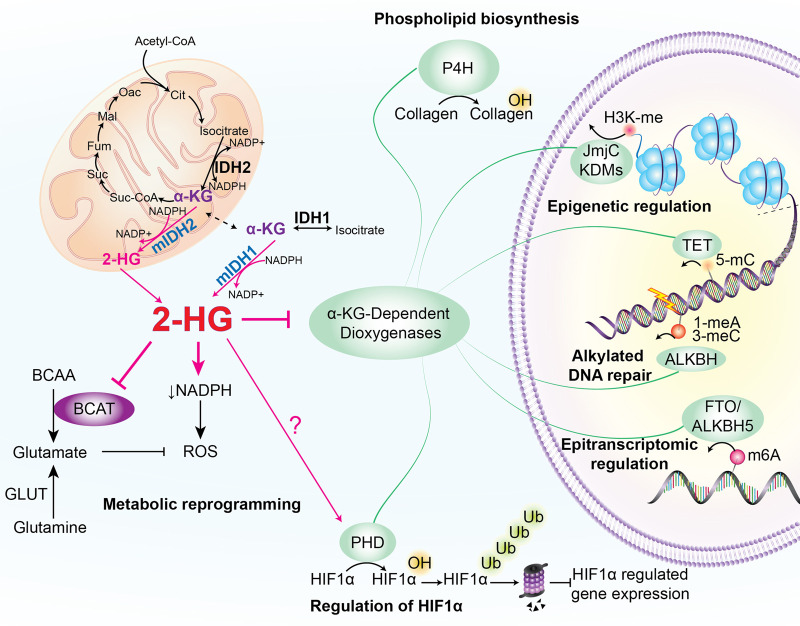
Pleiotropic effects of 2-HG. In cancer, IDH-mutant cells reduce the TCA metabolite α-KG to 2-HG which accumulates to supraphysiological levels and dysregulates a multitude of pathways to promote increased self-renewal and differentiation arrest. 2-HG can inhibit α-KG-dependent dioxygenases which are involved in a broad range of cellular functions including epigenetic regulation (histone and DNA demethylation), alkylated DNA repair, epitranscriptomic regulation (m6A demethylation), phospholipid biosynthesis and HIF-1α regulation. 2-HG can also induce large metabolic reprogramming, such as an increased vulnerability to oxidative stress driven in part by the consumption of NADPH for 2-HG production and increased reliance upon glutamine metabolism for glutamate production.

Cancer-associated mutations in IDH1 and -2 are typically heterozygous and result in substitution of arginine residues within the enzymes’ active site (most commonly R132 of IDH1, or R140 or R172 of IDH2). Seminal studies by Dang et al. [4] revealed that these changes not only abrogate the oxidative activity of the enzyme, but also alter the conformation of the active site such that a partial ‘reverse’ reaction is favoured in which α-KG is reduced but not carboxylated to produce 2-HG and NADPH is consumed generating NADP^+^ (Figure 1). The increased production of 2-HG overwhelms the 2-HG dehydrogenase enzyme that converts this usually low abundance metabolite back to α-KG and consequently levels of 2-HG in IDH-mutant cells are increased by several orders or magnitude [4,5].

It is notable that different cancer-associated IDH mutations have varied capacity to produce 2-HG, as demonstrated in isogenic cell lines and mouse models [13,14]. Moreover, while increased 2-HG synthesis is common to both IDH1 and -2 mutations, other metabolic perturbations appear to be distinct and are linked with reduced wild-type IDH1 or -2 function, respectively [15,16]. Together, these factors underpin the different prevalence of specific IDH1 and -2 variants in different cancer types, the pattern of co-occurring mutations, response to therapy and prognosis [2,3,17].

### IDH mutations in leukaemia initiation and maintenance

Multiple *in vitro* and *in vivo* studies have now confirmed IDH mutations as bona fide oncogenes. *In vitro*, IDH-mutant proteins confer cytokine-independent growth, differentiation arrest and promote aberrant self-renewal in various experimental systems. Expression of mutant IDH has been reported to promote GM-CSF-independent growth of the erythroleukaemia cell line TF-1 [18]; block PMA induced differentiation of THP1 cells [19]; and promote serial replating capacity of primary haematopoietic stem cells [20]. Similar observations have also been made in non-haematopoietic cells [21,22]. Importantly, some of these effects can be re-capitulated by membrane-permeable 2-HG, suggesting that neomorphic enzymatic activity is at least a major if not the predominant driver of cellular transformation [18,21,22].

Additional insights into the oncogenic potential of IDH mutations have come from experiments using mouse models. Sasaki et al. [23] generated a conditional knock-in mouse that allowed for haematopoietic-specific expression of IDH1R132H from the endogenous locus. Although the knock-in mice did not develop leukaemia, they did demonstrate a pre-malignant phenotype characterised by a partial block of differentiation in early multi-potent progenitors, anaemia, and extramedullary haematopoiesis. Subsequent work using different modelling strategies confirmed that although IDH mutations were insufficient on their own to transform primary cells, they could co-operate with other genetic events to initiate tumourigenesis [20,24–27]. These studies are consistent with the observation that IDH mutations co-occur with other genetic lesions in AML and in human cancer more broadly [2,3,17,28].

Not only do IDH mutations play a role in AML initiation, but transformed leukaemic cells appear to require continued 2-HG production for proliferation and survival in certain contexts. Using inducible multigenic AML models, we and others have found that silencing mutant IDH in the context of multiple strong leukaemic driver genes has a profound impact on tumour maintenance [20,26,29,30]. While these studies strongly support the case for IDH inhibitors as anti-AML therapies, it should be noted that the molecular and cellular effects of 2-HG depletion are complex and highly context dependent. Normalisation of the 2-HG-induced aberrant epigenetic state occurs over a period of time and may be incomplete [27,31]. Moreover, the functional significance of mutant IDH for self-renewal and differentiation of a particular cell is influenced by co-occurring mutations and epigenetic factors [30,32–34]. Ultimately, this leads to a complex pattern of response and resistance to IDH inhibition in AML as discussed in more detail below.

### Molecular targets of 2-HG

In addition to being a critical TCA metabolite, α-KG is an essential co-substrate for a functionally diverse group of enzymes, collectively termed α-KG-dependent dioxygenases, which are involved in epigenetic and metabolic regulation (Figure 1). Given the supraphysiological accumulation of 2-HG in IDH-mutant cells, 2-HG competitively outcompetes α-KG in the substrate-binding pocket of α-KG-dependent dioxygenases, despite α-KG-dependent dioxygenases having a lower binding affinity for 2-HG than α-KG [35–38]. Indeed, 2-HG levels are proportional to the perturbation of α-KG-dependent pathways [22,35,39–41], which can influence malignant transformation [13,18,39]. When compared with IDH2R140Q, IDH2R172K cells exhibit greater 2-HG accumulation, increased dysregulation of the epigenetic landscape and reduced disease latency *in vivo* [13,14,39]. Moreover, IDH2R172K AMLs typically harbour fewer and distinct co-occurring mutations than the IDH2R140Q sub-type [17,42], implicating the level of 2-HG production as a key driver for leukaemogenesis.

Arguably the most widely studied molecular targets of 2-HG are the α-KG-dependent dioxygenases involved in epigenetic regulation. Collectively, the JmjC histone lysine demethylases (KDMs) and the TET family of DNA demethylases represent the most abundant group of α-KG-dependent dioxygenases [35,43]. Several *in vitro* studies have demonstrated that 2-HG inhibition of TETs and JmjC KDMs results in the increased abundance and abnormal distribution of DNA and histone methylation, respectively [18,22,23,31,35,38,40,44,45]. It is proposed that these changes in chromatin architecture contribute to the pathogenesis of mutant-IDH driven AML by promoting self-renewal and repressing differentiation transcriptional networks [18,22,39,40,46]. For example, overexpression of IDH2R140Q in an erythroleukaemia cell line (TF-1) resulted in hypermethylation of DNA and repressive histone marks, which could be reversed upon mutant-IDH inhibition [40]. Specifically, under conditions that typically promote erythroid differentiation, the expression of IDH2R140Q in TF-1 cells increased DNA methylation and H3K9me3 at the promoter of a key differentiation gene, *HBG*, suppressed *HBG* expression and blocked differentiation, all of which were reversed upon inhibition of mutant-IDH [40]. Aberrant DNA and histone methylation have also been reported in mutant-IDH expressing haematopoietic stem and progenitor cells *in vivo* [23,39] and in numerous studies where 2-HG is supplemented or mutant-IDH expressed in non-haematological malignancies [22,31,35,45,47–51]. Moreover, analyses of AML patient samples have shown that DNA hypermethylation in TET2-mutant AMLs overlaps with the hypermethylation phenotype of IDH-mutant AML [44,52,53]. Additionally, TET2- and IDH-mutant AMLs exhibit significantly reduced 5hmC levels within IDH-mutant DNA hypermethylated regions when compared with AML patient samples that were wild-type for TET2 and IDH [53], collectively suggesting that the effects of mutant-IDH on DNA methylation are driven in part by 2-HG inhibition of TET2. Interestingly, despite the overlap in the TET2- and IDH-mutant DNA methylation phenotype, the DNA methylation patterning in mutant-IDH AML patient samples is largely distinct from all other AMLs, including TET2-mutant AML [44,52]. This is thought to be in part due to the modest DNA hypermethylation phenotype in TET2-mutant AMLs relative to IDH-mutant AMLs, which may be exacerbated in IDH-mutant AMLs as 2-HG can inhibit: (1) other TET enzymes [35] and; (2) JmjC KDMs resulting in increased H3K9 and H3K36 methylation [23,40] that in turn can recruit complexes to modify the DNA methylome [54,55]. This highlights the challenges in delineating how 2-HG inhibition on individual α-KG-dependent epigenetic regulators directly contributes to leukaemogenesis, as the simultaneous 2-HG inhibition of multiple epigenetic regulators likely acts both additively and antagonistically to reshape the epigenome and promote malignant transformation. Although collectively these studies provide evidence that 2-HG driven changes to the chromatin architecture are important for oncogenesis, few studies have directly correlated mutant-IDH driven changes of the histone methylome with gene expression in AML and, to date, no studies have examined the histone methylation patterns in IDH-mutant AML patient samples.

2-HG can also inhibit members of the alkylated DNA repair AlkB homologue (ALKBH) family of proteins which utilise α-KG as a co-substrate to remove mutagenic and cytotoxic methylated lesions (e.g. 1-meA, 3-meC) caused by alkylating agents [56], and demethylate N6-methyladenosine (m6A), which is the most prevalent RNA modification in eukaryotes and is involved in splicing and translational control of gene expression [57–59]. ALKBH2 and ALKBH3, required for repairing alkylated DNA, are inhibited by 2-HG which results in the accumulation of 1-meA and 3-meC in single-and double-stranded DNA and increased DNA damage [60,61]. IDH mutations are frequently found in premalignancies, such as MDS, and are considered an early event in leukaemogenesis [62–65]. Thus, increased DNA damage by 2-HG inhibition of ALKBH2/3 may aid in leukaemogenesis through the accumulation of mutations. Mutant-IDH cell lines and AML patient samples also display elevated m6A levels [58,66,67]. Interestingly, recent studies postulate an anti-tumour effect of 2-HG, whereby 2-HG inhibition of FTO, the primary enzyme responsible for m6A demethylation, promotes transcriptional and metabolic changes that underpin the survival advantage that mutant-IDH glioma patients have relative to wild-type IDH patients [58,67–69], although this survival trend appears to be debatable in AML [70–73]. However, these studies did not utilise models that express mutant-IDH and are inherently dependent upon 2-HG for leukaemogenesis, proliferation or maintaining leukaemic capacity. Although elevated m6A has been reported in mutant-IDH patient samples, further mechanistic studies are required to understand how this contributes to mutant-IDH pathogenesis.

Hypoxia-inducible factor α (HIF-α) is regulated by α-KG-dependent HIF prolyl hydroxylases (PHD/EGLN); under normoxic conditions, HIF-α is hydroxylated by PHDs and targeted for proteasomal degradation, whilst hypoxia reduces PHD activity enabling translocation of HIF-α to the nucleus and expression of HIF-α targets [74]. Despite being an α-KG-dependent dioxygenase, the impact of 2-HG on PHDs remains poorly understood in AML, and controversial in the context of mutant-IDH glioma. Initial studies in glioma cell lines reported the expected 2-HG inhibitory effects on PHDs and consequent elevated HIF-1α levels; overexpression of mutant-IDH, down-regulation of wild-type IDH1 or exogenous 2-HG supplementation up-regulated HIF-1α, whilst concurrent exogenous supplementation of α-KG with 2-HG reversed the 2-HG-induced increase in HIF-1α [35,75]. However, several studies have provided conflicting evidence whereby mutant-IDH reduced HIF-1α expression by the paradoxical 2-HG-mediated activation of PHDs in glioma and haematopoietic cells, and PHD knockdown conferred a proliferative disadvantage in mutant-IDH cells [18,21]. These data suggest that 2-HG acts as an agonist of PHDs thereby reducing HIF-1α levels and promoting tumourigenesis, which is concordant with the repression of HIF-1α targets in IDH-mutant, relative to wild-type, glioma patients [76]. However, suppression of HIF-1α targets was not observed in mutant-IDH expressing haematopoietic stem cells *in vivo* [23]. Overall, additional research is required to elucidate the impact of 2-HG on PHDs and the contribution to leukaemogenesis.

In addition to the α-KG-dependent dioxygenases aforementioned, 2-HG can also inhibit the α-KG-dependent transaminases BCAT1 and BCAT2 [41]. Branched-chain amino transferases (BCATs) initiate the catabolism of branched-chain amino acids (BCAAs), including leucine, isoleucine and valine, via transamination of BCAAs to branched-chain α-ketoacids (BCKA) coupled with the simultaneous interconversion of α-KG to glutamate [77]. *In vitro* studies in mutant-IDH glioma cells found that 2-HG inhibition of BCAT1/2 resulted in elevated BCAAs levels and concurrent decreased BCKAs and glutamate [41,78]. This rendered IDH-mutant cells dependent on glutamine metabolism for glutamate production. Cells require glutamate as a nitrogen donor for the synthesis of metabolites vital for proliferation, and the production of glutathione which aids to resist oxidative stress by the removal of reactive oxidative species (ROS). Thus, these observations highlight a potential synergistic interaction between mutant-IDH inhibitors and glutaminase inhibitors. The increased vulnerability to oxidative stress and other metabolic dependencies in IDH-mutant cells stems from the large metabolic reprogramming induced by 2-HG which has been extensively reviewed elsewhere [79]. For example, mutant-IDH cells consume large amounts of NADPH for 2-HG synthesis, thereby reducing NADPH levels and competing with NADPH-dependent pathways including redox homeostasis and reductive biosynthesis of lipids [80–82]. Relative to wild-type IDH glioma cells, mutant-IDH cells have reduced glycolysis and impaired TCA cycle function, thus rendering the cells more dependent on oxidative phosphorylation [83–85]. Mutant-IDH cells also exhibit reduced phospholipid biosynthesis relative to wild-type IDH malignant cells through 2-HG inhibition of the α-KG-dependent dioxygenase collagen-4-prolyl hydroxylase (P4H) [86,87]. Of note, these studies were largely undertaken in glioma models, and thus the extent of metabolic reprogramming and contribution to pathogenesis in mutant-IDH AMLs remains to be elucidated.

Given the myriad enzymes that are inhibited by 2-HG, it has been difficult to identify any individual protein as the major or dominant driver of the mutant IDH phenotype. Not surprisingly, the capacity of 2-HG to inhibit particular enzymes *in vitro* varies significantly [88], although as diverse assays are required to assess different enzymatic activities, direct head-to-head comparisons should be made with caution. The situation is also likely to be extremely complex *in vivo*, with a multitude of factors including relative expression and cellular localisation, co-occurring genetic lesions, differentiation and metabolic states likely affecting the relative inhibition of specific proteins. Thus, while individual molecular changes can play more or less prominent roles in specific contexts, it is the combinatorial effect of mutant IDH proteins on multiple pathways that hold the key to their overall capacity to drive cancer.

### Paracrine effects of 2-HG on the tumour microenvironment

In addition to its cell-intrinsic effects, the high levels of 2-HG that accumulate in the tumour microenvironment have the potential to exert paracrine effects on IDH wild-type cells that co-occupy the same niche. In the context of glioma, tumour-derived 2-HG is taken up by infiltrating T-lymphocytes, negatively impacting anti-tumour immunity [89,90]. Although similar findings have not been reported in AML to date, it appears highly likely that 2-HG similarly affects the immune landscape in this disease. Beyond T-cells, 2-HG could conceivably affect many other cell types with implications for disease progression and therapeutic response. This includes other immune and stromal cells, as well as IDH wild-type malignant clones that may be present (AML patients often possess upwards of five malignant and pre-malignant clones at once [28]). Indeed, extracellular 2-HG has been reported to activate NF-κB-dependent transcriptional signatures in stromal cells; immortalised human bone marrow cells supplemented with 2-HG or conditioned media from mutant-IDH AML cells activated NF-κB and downstream genes, including cytokine genes that promote AML proliferation, which was concordant with the transcriptional profiles from primary bone marrow stromal cells isolated from mutant-IDH AML patients [91]. These data suggest that extracellular 2-HG may provide a supportive microenvironment for leukaemogenesis. Although 2-HG is generally considered to be poorly cell permeable, several investigators have reported that ‘unmodified’ 2-HG (i.e. chemically identical with what is produced by IDH-mutant cells and distinct from the cell-permeable versions used in most studies) administered *in vitro* or *in vivo*, albeit at very high levels, can enter different cell types and impact their function [58,92]. These studies point to the intriguing possibility that IDH-mutant AML cells could either promote or suppress the survival of other malignant clones.

## Therapeutic targeting of IDH-mutant AML

### IDH inhibitors

The distinct catalytic site responsible for the neomorphic activity of mutant-IDH coupled with the evidence that mutant-IDH is important for AML pathogenesis, prompted rapid drug discovery to target mutant-IDH. Ivosidenib and Enasidenib are FDA approved allosteric inhibitors of mutant-IDH1 and IDH2, respectively, that decrease serum 2-HG levels, induce differentiation of leukaemic blasts and achieve complete remission irrespective of haematological recovery in over 30% of relapsed/refractory and newly diagnosed *IDH*-mutant AML patients [42,93–98]. Studies in pre-clinical models have demonstrated that mutant-IDH inhibitors reverse the 2-HG mediated cellular changes including DNA and histone hypermethylation, which is accompanied by vast transcriptional changes that abrogate self-renewal and promote differentiation of leukaemic stem cells [20,26,27,29,40,93]. However, as with most AML therapies, primary and acquired resistance to small-molecule inhibitors of mutant-IDH is not uncommon and mechanistically poorly understood for the majority of patients. Neither serum 2-HG levels, mutant-IDH variant allele frequencies, nor DNA demethylation are prognostic markers of durable response in patients [33,42].

Case studies have reported patient-acquired resistance to IDH inhibitors by maintaining 2-HG dependence through mutations that restore 2-HG levels including isoform switching, whereby mutations arise in the IDH homologue that was initially free of genetic lesions and not the target of therapy [99], and second-site mutations at the IDH dimer interface that interfere with IDH inhibitor binding [100,101]. The latter was reported to occur both *in cis* and *in trans*, such that resistance mutations arose in the IDH allele with or without the initial neomorphic mutation, respectively [100]. More recently, an *in vitro* study utilising CRISPR base-editing screens in IDH1- and IDH2-mutant leukemic cells uncovered a catalogue of IDH second-site mutations that confer resistance to targeted therapies [102]. In addition to second-site mutations at the dimer interface, the authors identified resistant mutations in IDH that interfere with NADPH binding and confirmed their presence in cases of IDH2R140Q mutant AML patients that developed resistance to Enasidenib [102]. The acquisition of resistance mutations that restore 2-HG levels, and thus maintain 2-HG dependence, has been reported in ∼25% of IDH1-mutant AML patients that developed resistance to Ivosidenib [101], although others report lower incidence rates [32,33], highlighting the need for more studies with larger cohort sizes to better ascertain the true prevalence of isoform switching and second-site mutations.

Co-occurring mutations in common AML drivers have also been associated with poorer clinical outcomes. Targeted sequencing has implicated activating mutations in the receptor tyrosine kinase (RTK) pathway with both primary and acquired resistance to IDH inhibitors [33,42,94,95,101], although the underlying mechanism remains elusive. Co-occurring mutations in transcription factors that regulate haematopoiesis and myeloid differentiation, including *RUNX1, CEBPA* and *GATA2*, have been associated with poorer clinical outcomes to IDH inhibitors [32,33,101]. It is hypothesised that mutations in haematopoietic transcription factors contribute to resistance by attenuating differentiation programs induced by IDH inhibitors [33], although this has yet to be validated. Additionally, loss-of-function mutations in BCOR and TET2 have been associated with acquired resistance to IDH inhibitors [32,33,101]. Wang and colleagues reported the emergence of TET2 mutations at relapse with a concomitant de-repression of DNA methylation, despite maintained suppression of 2-HG [33], suggesting that maintenance of the hypermethylation phenotype is important for leukaemia survival in a subset of patients.

Leukaemia stem cells display increased self-renewal capacity and transcriptional plasticity, and are largely resistant to many cytotoxic drugs [103–105]. Multi-logistic regression analysis found that increased leukaemia stemness, derived from the LSC17 score [105], is associated with primary resistance to IDH inhibitors [33]. Moreover, the authors reported that increased stemness was associated with baseline promoter hypermethylation, which was correlated with the repression of haematopoietic differentiation genes and the absence of *DNMT3A* mutations [33]. Although mechanistic understanding of the drivers of stemness in mutant-IDH AML are still lacking, these data suggests that leukaemia stemness signatures underscore a potential prognostic biomarker for IDH inhibitor response.

Adding to the complexity of response to IDH inhibitors, Quek et al. [32] reported that clonal heterogeneity greatly influences IDH inhibitor response. At baseline, the authors report marked inter-patient variation in the expanded leukaemic compartments that was dependent on the co-occurring mutations within the clonal architecture, suggesting that the degree of differentiation arrest by mutant-IDH2 is context dependent [32]. Patients that entered complete remission following Enasidenib treatment exhibited restoration of normal cell-type proportions within the haematopoietic compartment, although, the IDH2-mutant clone was not eradicated in most patients [32]. Clonal evolution or selection drove acquired resistance to Enasidenib by restoring the differentiation arrest [32]. However, further studies are required to better understand the molecular mechanisms, including the epigenetic and transcriptional remodelling, that underpin the clonal response to IDH inhibitors.

### Synthetic lethal and combination strategies

To improve the clinical outcomes of mutant-IDH AML patients, several clinical trials were initiated to investigate rational IDH inhibitor combinations. A randomised phase 3 clinical trial is currently underway to assess the combination of IDH inhibitors with induction, consolidation and maintenance therapies in newly diagnosed IDH-mutant AML patients that are eligible for intensive chemotherapy (NCT03839771). However, many AML patients are ineligible for standard of care intensive chemotherapy and are instead treated with hypomethylating agents (HMAs), which are well-tolerated and thus improved clinical outcomes are frequently assessed by combining HMAs with targeted AML therapies [106]. HMAs, including 5-Azacytidine (AZA) and Decitabine, are cytidine analogues that inhibit the DNA methyltransferase activity of DNMT1 which results in DNA hypomethylation [106]. The AGILE study, a phase 3 clinical trial, reported a significantly longer event-free survival when Ivosidenib was given in combination with AZA, relative to AZA monotherapy, in newly diagnosed IDH1-mutated AML patients that were ineligible for intensive chemotherapy induction [107]. Moreover, higher overall response rates have been reported for the combination of AZA and IDH inhibitors [107,108] in comparison with IDH inhibitor monotherapies [93,94,97,98]. Pre-clinical *in vivo* studies suggest that the synergy of AZA and IDH inhibitor combination is due to rapid depletion of leukaemia stem cells driven by: (1) the IDH-inhibitor induced up-regulation of gene networks required for HMA efficacy, including cell-cycling and pyrimidine salvage; and (2) the enhanced transcriptional response, including increased myeloid differentiation and self-renewal repression, induced by the combination relative to the single-agents [29,109].

Other therapeutic approaches that seek to target genes that are synthetic lethal with IDH mutations are also being explored. Among the first synthetic lethal partners of mutant IDH to be described was the anti-apoptotic protein BCL2. The relationship between BCL2 and mutant IDH proteins was initially uncovered in an elegant RNAi screen using syngeneic IDH-mutant and wild-type leukaemic cells [110]. BCL2 knockdown cells were selectively depleted in the IDH-mutant context and treatment of wild-type cells with 2-HG sensitised cells to BCL2 silencing. IDH-mutant cells were also shown to be more sensitive to the BCL2 inhibitor Venetoclax, an observation that was later validated in patients [111]. The increased efficacy of Venetoclax was attributed to the inhibition of cytochrome *c* oxidase (aka complex IV of the mitochondrial electron transport chain) by 2-HG, which lowered the threshold for induction of the intrinsic apoptosis pathway [110]. While the sensitivity of IDH-mutant cells to BCL2 inhibition *in vitro* is dependent on 2-HG [110], the situation appears to be more complex *in vivo* with the combination of IDH inhibitors and Venetoclax (administered concurrently) demonstrating superior anti-leukaemia efficacy in mouse models and early clinical trials [112,113]. Whether this is explained by pharmacodynamic differences *in vivo*, or because IDH inhibitors and Venetoclax target different cell types in genetically and epigenetically heterogenous AML tumours remains under investigation.

More recently, 2-HG has been found to induce a severe homologous recombination defect by inhibiting the histone demethylase KDM4B, leading to aberrant hypermethylation of histone 3 lysine 9 (H3K9) and defective signalling at DNA damage foci [48,114]. The DNA damage response is further compromised by local H3K9 hypermethylation and down-regulation of the key DNA damage sensor ATM [115]. The reduced capacity of IDH-mutant cells to repair their genomes via homologous recombination, renders them reliant on PARP-mediated DNA repair, and results in a BRCAness-like sensitivity to PARP inhibitors [116].

Numerous other IDH-mutant synthetic lethal interactions have also been suggested, although these remain at the preclinical stage for now. IDH1 (but not IDH2) mutant leukaemic cells demonstrate increased sensitivity to mitochondrial electron transport chain complex I inhibitors [117], and an increased dependence on the lipid synthesis enzyme ACC1 and β-oxidation of fatty acids [16]. Notably the latter potentially points to the exciting possibility that dietary modifications could be used to enhance the efficacy of anti-cancer therapies. IDH-mutant glioma cells were recently found to be hypersensitive to inhibitors of *de novo* pyrimidine biosynthesis [118]. Whether this is also the case in AML is currently unclear, although AML cells (irrespective of IDH-mutant status) are known to be highly reliant on *de novo* pyrimidine synthesis [119].

## Concluding remarks

AML is a poor prognosis cancer and there is an urgent unmet need for new personalised medicine approaches to combat tumour heterogeneity and drug resistance. IDH mutations represent attractive therapeutic targets, with multiple avenues for selective elimination of IDH-mutant cells. Delineating resistance mechanisms to IDH inhibitors, particularly in the subset of patients that fail to clear IDH-mutant leukaemic clones despite effective suppression of 2-HG, remains a major challenge for the field. Likewise, understanding the paracrine effects of 2-HG on the tumour microenvironment is an area of research that requires further effort. Combination strategies, including with HMAs and Venetoclax, are showing significant promise [107,113]. On current evidence, it should be possible to permanently extinguish IDH-mutant AML in most patients for whom IDH mutations occur as one of the early lesions in disease evolution.

## Perspectives

- Somatic mutations in IDH1 and -2 occur in several cancers including AML. Production of the oncometabolite 2-HG by mutant IDH proteins along with loss of wild-type IDH activity contributes to leukaemia initiation and maintenance.
- High levels of 2-HG in IDH-mutant cells dysregulates a multitude of cancer-related pathways. Most notably, 2-HG inhibits a class of enzymes termed α-KG dependent dioxygenases which includes proteins that control DNA and histone methylation and hypoxic signalling.
- Several strategies for selective targeting of IDH-mutant cells have emerged. Small molecule blockade of 2-HG is effective, but resistance via several pathways is commonplace. Pathways that are synthetic lethal with IDH mutations have also been identified. Further work is required to convert what are currently short- and medium-term responses into long-term remissions.

## Open Access

Open access for this article was enabled by the participation of University of Melbourne in an all-inclusive *Read & Publish* agreement with Portland Press and the Biochemical Society under a transformative agreement with CAUL.

## Abbreviations

2-HG: D-2-hydroxyglutarate
AML: acute myeloid leukaemia
AZA: 5-Azacytidine
BCAAs: branched-chain amino acids
HIF: hypoxia-inducible factor
HMAs: hypomethylating agents
IDH: isocitrate dehydrogenase
KG: ketoglutarate
PHD: prolyl hydroxylases
